# Deep proteome coverage advances knowledge of *Treponema pallidum* protein expression profiles during infection

**DOI:** 10.1038/s41598-023-45219-8

**Published:** 2023-10-25

**Authors:** Simon Houston, Alloysius Gomez, Andrew Geppert, Azad Eshghi, Derek S. Smith, Sean Waugh, Darryl B. Hardie, David R. Goodlett, Caroline E. Cameron

**Affiliations:** 1 Department of Biochemistry and Microbiology, University of Victoria, Victoria, BC Canada; 2 University of Victoria-Genome BC Proteomics Centre, University of Victoria, Victoria, BC Canada; 3 Division of Allergy and Infectious Diseases, Department of Medicine, University of Washington, Seattle, WA USA

**Keywords:** Proteomics, Bacteria, Pathogens

## Abstract

Comprehensive proteome-wide analysis of the syphilis spirochete, *Treponema pallidum* ssp. *pallidum*, is technically challenging due to high sample complexity, difficulties with obtaining sufficient quantities of bacteria for analysis, and the inherent fragility of the *T. pallidum* cell envelope which further complicates proteomic identification of rare *T. pallidum* outer membrane proteins (OMPs). The main aim of the present study was to gain a deeper understanding of the *T. pallidum* global proteome expression profile under infection conditions. This will corroborate and extend genome annotations, identify protein modifications that are unable to be predicted at the genomic or transcriptomic levels, and provide a foundational knowledge of the *T. pallidum* protein expression repertoire. Here we describe the optimization of a *T. pallidum*-specific sample preparation workflow and mass spectrometry-based proteomics pipeline which allowed for the detection of 77% of the *T. pallidum* protein repertoire under infection conditions. When combined with prior studies, this brings the overall coverage of the *T. pallidum* proteome to almost 90%. These investigations identified 27 known/predicted OMPs, including potential vaccine candidates, and detected expression of 11 potential OMPs under infection conditions for the first time. The optimized pipeline provides a robust and reproducible workflow for investigating *T. pallidum* protein expression during infection. Importantly, the combined results provide the deepest coverage of the *T. pallidum* proteome to date.

## Introduction

*Treponema pallidum* ssp. *pallidum* is the causative agent of syphilis. Increasing rates of infectious and congenital syphilis^1–5^, and the increased risk of HIV transmission and acquisition in symptomatic syphilis infections^6, 7^, highlight the need for the development of an effective syphilis vaccine to achieve global elimination of syphilis^8^.

In the absence of treatment, *T. pallidum* can persist within a host for decades^9^. Unconventional ultrastructural characteristics of the *T. pallidum* cell envelope, including an unusually low number of surface-exposed outer membrane proteins^10–12^, contribute to its “stealth pathogenicity”^10–14^. Since *T. pallidum* lacks surface structures that are frequently found in Gram-negative and Gram-negative-like bacteria (i.e. lipopolysaccharide, S-layers, etc.)^9, 15^, treponemal OMPs are one of the first lines of contact between the bacterium and the host during infection. Further, a subset of surface-exposed OMPs belonging to the 12-membered *T. pallidum* repeat (Tpr) protein family have been shown to undergo both antigenic^16–18^ and phase variation^19, 20^, allowing evasion of the immune response^9^. Overall, *T. pallidum* OMPs comprise critical targets for syphilis vaccine design.

Global proteomic analysis is regarded as an important approach for gaining insight into bacterial pathogenesis and the biology of pathogens via several approaches, including: (1) protein quantification analyses (in vivo- versus in vitro-cultured bacteria, clinical strains versus laboratory strains, pathogenic strains versus non-pathogenic strains etc.); (2) examination and inter-strain comparison of specific protein expression profiles (e.g. proteins pertaining to virulence [virulence factors]); (3) confirmation and inter-strain comparison of the expression of functionally-unannotated proteins and “hypothetical proteins” that may play important roles in microbial biology and pathogenesis^21, 22^; and (4) the identification of proteins that are essential for basic functioning and survival. The *T. pallidum* genome contains approximately 1000 predicted protein-coding genes^15^. Global proteomic analysis of *T. pallidum* is complicated by the fact that the bacterium is an obligate human pathogen that is routinely grown in rabbits (in vivo-grown *T. pallidum*) or in the presence of rabbit epithelial cells (in vitro-grown *T. pallidum*)^23^. Each of these growth conditions produce low numbers of bacteria for experimentation, and result in protein preparations that contain contaminating rabbit proteins at concentrations far in excess of the *T. pallidum* proteins, thus decreasing the chances for successful detection of lower abundance *T. pallidum* proteins via methodologies such as mass spectrometry analyses. In addition, the *T. pallidum* outer membrane is inherently fragile and easily disrupted by experimental manipulations due to the unusual ultrastructure of the cell envelope^11, 12^. This presents an extra complication for proteomic confirmation of expression of rare *T. pallidum* OMPs that are readily lost due to shearing of the outer membrane. Despite these challenges, two previous whole proteome profiling studies identified a combined total of 587 *T. pallidum* proteins in experimental rabbit infections (in vivo-grown *T. pallidum*), representing approximately 60% coverage of the treponemal proteome^24, 25^.

In the present study, the major aim was to extend the coverage of the *T. pallidum* proteome. This was achieved via the optimization of a treponemal-specific proteomics workflow that circumvents the technical issues associated with experimental manipulation of *T. pallidum* and subsequent proteomic analyses in the presence of high amounts of contaminating rabbit proteins. This approach allowed: (1) achievement of the most comprehensive *T. pallidum* proteome coverage to date, with detection of the majority of the predicted/known OMP repertoire, including several OMPs that are being pursued as syphilis vaccine candidates; and (2) identification of genome/proteome annotation inaccuracies that erroneously exclude expressed *T. pallidum* proteins and mis-identify sites of protein translation initiation. The optimized workflow and resultant enhanced proteome coverage provides the opportunity for deep mining of the *T. pallidum* proteome, improved knowledge of the protein complement expressed by *T. pallidum* that is responsible for the novel biology and pathogenesis of this bacterium, and protein expression information under infection conditions that is relevant to syphilis vaccine development.

## Methods

### Propagation and isolation of *T. pallidum*

*Treponema pallidum* subsp. *pallidum* (Nichols strain) was propagated in male specific pathogen-free (SPF) New Zealand White rabbits as described elsewhere^26^. The animal study was reviewed and approved by the local institutional review board at the University of Victoria under protocol 2020-024, and was conducted in strict accordance with standard accepted principles as set forth by the Canadian Council on Animal Care, National Institutes of Health and the United States Department of Agriculture in a facility accredited by the Canadian Council on Animal Care and the American Association for the Accreditation of Laboratory Animal Care. For extraction, treponemes were harvested from the testes of rabbits approximately 10–12 days after infection in sterile saline (0.9% w/v NaCl, pH 7.0) in the presence or absence (refer to method development section below for details) of 10% normal rabbit serum (NRS). Extractions were performed in an anaerobic chamber (Coy Laboratories, Grass Lake, MI, USA) at room temperature in an atmosphere of 1.5–3% O_2_ and 5% CO_2_, balanced with N_2_. Rabbit cells and debris were separated and removed as described below. Treponemes in suspension were enumerated using a Nikon Eclipse E600 darkfield microscope (Nikon Canada, Mississauga, ON, Canada).

### *Treponema pallidum* protein sample preparation; method optimization

**Figure 1:**
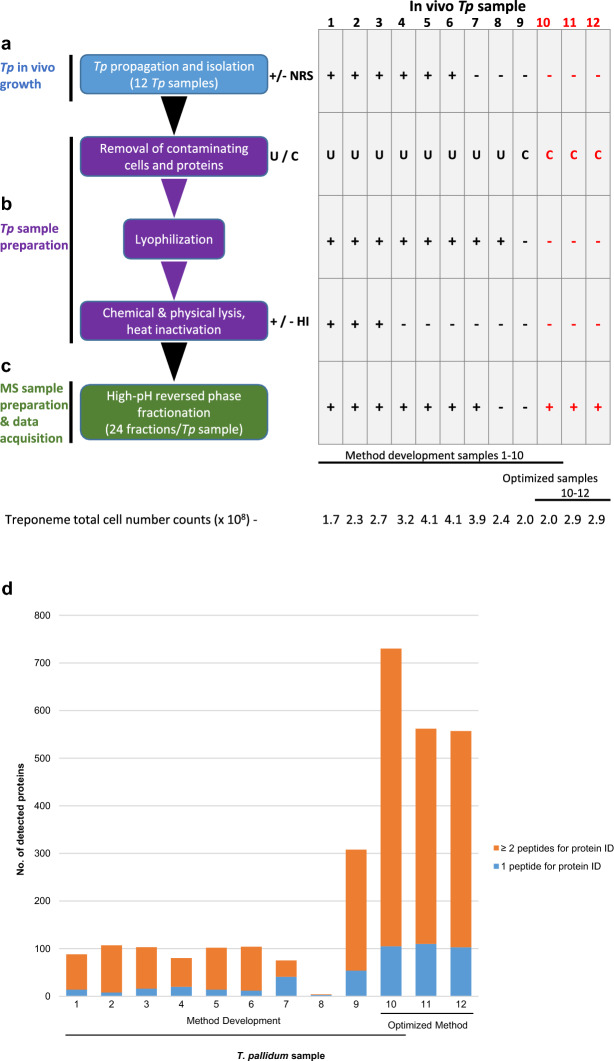
Optimization of a workflow for deep proteome coverage of in vivo-grown *T. pallidum*. Schematics showing (**a**) isolation of *T. pallidum* from rabbits, (**b**) the major steps comprising *T. pallidum* sample preparation, and (**c**) the main optimization step in mass spectrometry sample preparation, processing, and data acquisition. The workflow indicates each of the individual steps that were performed in the optimization of the protocol for global profiling of *T. pallidum* protein expression (left). The corresponding table shows the variable parameter conditions used in each of the biological replicate samples at each of the individual optimization steps (right). Samples 1–10 were used for optimizing the protocol during the method development stages; sample 10 conditions were found to be optimal (red text). Samples 11 and 12 (red text) correspond to two biological replicate samples that were processed using the optimized protocol used for sample 10 to obtain three biological replicate samples prepared using an identical protocol. The total number of treponemes used in the preparation of each of the 12 samples is indicated (bottom). Plus sign; a parameter condition has been included in a protocol step; minus sign, a parameter condition has been omitted in a protocol step. +/− NRS = the addition or omission of normal rabbit serum (NRS) during *T. pallidum* isolation. U/C = the use of either ultrafiltration (U) or high-speed centrifugation (C) during removal of contaminating rabbit components. +/− HI = the use or omission of heat inactivation during *T. pallidum* sample preparation. (**d**) The total number of *T. pallidum* proteins that were detected and identified in each of the 12 in-vivo grown *T. pallidum* biological replicate samples. For each bar, the number of *T. pallidum* proteins that were identified via the detection of one tryptic peptide (blue) or two tryptic peptides (orange) in each of the 12 individual samples is indicated.

Due to the inherent fragility of *T. pallidum* and the sample complexity arising from contaminating rabbit proteins/cellular debris from in vivo culturing of *T. pallidum*, we optimized a protocol for the preparation and mass spectrometry-based analyses of *T. pallidum* samples in order to maximize coverage of the *T. pallidum* proteome. The flow diagram shown in Fig. 1 outlines all major steps and parameters that were tested in the optimization steps, including the isolation of *T. pallidum* (as described above) and mass spectrometry sample preparation (as detailed below). Since some of the key methods used in our *T. pallidum* proteomics workflow had not been used in previous treponemal proteomics studies, there were limited literature reports that could inform workflow optimization. In our initial optimizations (samples 1–9), we used methods from previous proteomics analyses of other organisms. In our later optimization analysis (sample 10), we combined all the methods from our initial findings (samples 1–9) that had led to increased *T. pallidum* proteome coverage.

*Treponema pallidum* was isolated from rabbits (treponeme cell number range: 1.7–4.1 × 10^8^). The in vivo samples were harvested in either the presence (six samples) or absence (four samples) of NRS (Fig. 1a). NRS helps to maintain treponeme viability, however, its addition also increases the complexity of the samples by increasing the amount of contaminating rabbit proteins, including highly abundant albumin.

The next parameters to be investigated pertained to sample preparation of harvested *T. pallidum* (Fig. 1b). The first step in sample preparation involved removing as much contaminating rabbit cellular debris and proteins as possible. Two methods, each comprised of two components, were investigated; (1) low-speed centrifugation followed by ultrafiltration (eight samples), and (2) low-speed centrifugation followed by high-speed centrifugation (two samples). In both methods, insoluble rabbit gross cellular debris was removed via two centrifugation steps at 220 × *g* (5 min each, room temperature) followed by two additional centrifugation steps at 400 × *g* (7 min each, room temperature). In method (1), the slow-speed centrifugation steps were followed by ultrafiltration in order to remove soluble contaminants in the treponemal supernatant and to wash the suspended treponemes. In this method, the *T. pallidum*-containing supernatants (2.5 mL) were centrifuged at 220 × *g* (room temperature) using Protein Ark Proteus-X-Spinner 2.5 ultrafiltration concentrators (300 kDa MWCO) (Canadian Life Science, Peterborough, ON, Canada) until 1 mL of the *T. pallidum*-containing supernatants (retentate) remained in the bottom of the ultrafiltration concentrators. The ultrafiltrates were removed and 1.5 mL of sterile saline (0.9% w/v NaCl, pH 7.0) was added to wash the *T. pallidum*-containing retentate. The ultrafiltration wash/concentration steps were repeated two more times. The final concentrated and washed 1 ml T*. pallidum*-containing saline suspensions were pooled for each of the individual rabbits and stored at – 80 °C for subsequent lyophilisation. In method (2), the slow-speed centrifugation steps were followed by high-speed centrifugation in order to remove soluble contaminants in the treponemal supernatant and to wash the suspended treponemes. In this method, the *T. pallidum*-containing supernatants were centrifuged at 17,000 × *g* for 5 min at room temperature. The supernatant was discarded, and the *T. pallidum* pellet was gently resuspended in sterile saline (0.9% w/v NaCl, pH 7.0) and centrifuged again at 17,000 × *g* for 5 min at room temperature, after which the supernatant was discarded.

As shown in Fig. 1b, the eight *T. pallidum* samples that were subjected to ultrafiltration were lyophilized to concentrate the samples for mass spectrometry analyses. Following removal from − 80 °C storage, the frozen samples were lyophilized overnight (> 16 h) using a VirTis Freezemobile freeze dryer/lyophiliser (model 12EL; SP Industries, Warminster, PA, USA).

Chemical lysis of all 10 *T. pallidum* samples was performed by resuspending and incubating the lyophilized and pelleted samples in lysis buffer (500 µL per sample; 50 mM ammonium bicarbonate pH 8.0, 0.9% sodium deoxycholate [Sigma-Aldrich Canada Co., Oakville, ON, Canada]) for 30 min on ice, with 30 s vortex mixing steps every five min (Fig. 1b). Physical lysis was then performed on all 10 samples by ultrasonication at 6 °C using a Covaris ME220 focused-ultrasonicator with the following parameters: 3 × 30 s at 6W, 20% duty factor, 200 cycles per burst, 30 s rest between the three cycles (Covaris, LLC., Woburn, MA, USA).

Following cell lysis, three of the 10 samples were heated at 95 °C for 5 min in order to inactivate proteases and prevent non-trypsin mediated proteolysis of *T. pallidum* proteins (Fig. 1b). However, this resulted in heavy precipitation in the three samples. These three samples were recovered by centrifugation at 17,000 × *g* for 10 min. The supernatants were separated from the pellets and stored at 4 °C. The three pellets were incubated with solubilisation buffer (500 µL per sample; 300 mM Tris pH 8.0, 8 M Urea) for 45 min at room temperature with 60 s vortex mixing steps every 15 min. The three samples were then centrifuged at 13,000 × *g* for 10 min and the three urea-extracted supernatants were stored until trypsin digestion, as described below. In order to ensure complete removal of precipitated, aggregated, or insoluble proteins and cellular debris, all 10 T*. pallidum* lysate samples were centrifuged at 17,000 × *g* for 10 min at 4 °C. The supernatants were removed and protein concentrations were determined by measuring the absorbance at 280 nm (Beckman Coulter DU 730 Life Science UV/Vis Spectrophotometer; Beckman Coulter Canada, Mississauga, ON, Canada) and by performing BCA assays using the Thermo Scientific Pierce BCA protein assay kit (Thermo Fisher Scientific, Ottawa, ON, Canada).

### In solution trypsin digestion

Following the *T. pallidum* sample preparation optimization steps, all 10 lysate samples were digested with trypsin. For the three samples containing urea, 50 mM ammonium bicarbonate (pH 7.8) was added to 300 µg of protein (400 µL final volume). For samples containing no urea, 50 mM ammonium bicarbonate (pH 7.8) was added to obtain protein concentrations of ~ 1.7 mg/mL. Each sample was reduced at 37 °C for 30 min by the addition of 100 mM dithiothreitol (80 µL [urea-containing] or 120 µL [no urea samples]) and then alkylated at room temperature in the dark for 30 min by the addition of 240 mM iodoacetamide (80 µL [urea-containing] or 120 µL [no urea samples]). Urea-containing samples were diluted by the addition of 50 mM ammonium bicarbonate (pH 7.8) (2.6 mL). For the urea-containing samples, in-solution tryptic digestion was performed at 37 °C for 18 h by the addition of 30 µg of trypsin (Worthington Biochemical Corporation, Lakewood, NJ, USA). For the urea-free samples, 100 µg of trypsin was added per mg of protein. Protein digestion was stopped by the addition of formic acid (1% final concentration). Solid phase extraction cleanup was performed using Hydrophilic-Lipophilic-Balanced (HLB) columns (Waters Corporation, Milford, MA, USA). The digested protein samples were eluted with 60% acetonitrile/0.1% formic acid (300 µL per sample) and reduced to dryness in a SpeedVac vacuum concentrator. Samples were then re-suspended in 10 mM ammonium hydroxide, pH 10.0 (900 µL).

### High-pH reversed phase fractionation

To determine the effect of sample complexity reduction on whole proteome coverage, eight of the 10 trypsin-digested *T. pallidum* samples were separated into 24 fractions based on hydrophobicity using high-pH reversed phase fractionation (Fig. 1c). Specifically, an Agilent 1290 HPLC system (Agilent, Santa Clara, CA, USA) was equipped with an XBridge BEH300 C18 peptide separation technology (PST) column (250 mm × 4.6 mm, 5 µm, 300 Å) (Waters Corporation, Milford, MA, USA). Buffer A consisted of 10 mM ammonium hydroxide (pH 10.0), and buffer B was comprised of 80% acetonitrile and 10 mM ammonium hydroxide (pH 10). The *T. pallidum* samples were diluted to 0.9 mL total volume with buffer A and injected onto the column with a constant flow rate set at 0.75 mL/min. The column was equilibrated for 5 min in buffer A before a gradient (5–45%) of buffer B was performed over 75 min. Fractions were collected every min for 96 min, reduced in volume by lyophilisation, rehydrated with 2% acetonitrile/0.1% formic acid (300 µL), and concatenated into 24 fractions by combining every 24th fraction (e.g. fractions 1, 25, 49, and 73 were combined).

### LC–MS/MS analyses

The *T. pallidum* samples (fractionated and non-fractionated) were subjected to liquid chromatography-tandem mass spectrometry (LC–MS/MS) for global, high confidence protein identifications. An aliquot (5 µL) of each concatenated fraction or non-fractionated sample was separated by on-line reversed phase liquid chromatography using a Thermo Scientific EASY-nLC 1000 system with a reversed-phase pre-column packed with Magic C18-AQ resin (100 µm I.D., 2.5 cm length, 5 µm, 100 Å) and an in-house prepared reversed phase nano-analytical column packed with Magic C-18AQ resin (75 µm I.D., 15 cm length, 5 µm, 100 Å) (Michrom BioResources Inc., Auburn, CA, USA), at a flow rate of 300 nL/min. Solvent A was comprised of 2% acetonitrile and 0.1% formic acid while solvent B consisted of 90% acetonitrile and 0.1% formic acid. The *T. pallidum* samples were separated using a 120-min gradient comprised of the following steps: (1) 0–100 min, gradient change from 95% A/5% B to 58% A/42% B, (2) 100–115 min, gradient change from 58% A/42% B to 0% A/100% B, and (3) 115–120 min, gradient held at 0% A/100% B.

The chromatography system was coupled on-line with an Orbitrap Fusion Tribrid mass spectrometer (Thermo Fisher Scientific, San Jose, CA, USA) equipped with a Nanospray Flex NG source (Thermo Fisher Scientific). The Orbitrap Fusion Tribrid mass spectrometer instrument parameters (Fusion Tune 3.3 software) used were as follows: nano-electrospray ion source with spray voltage = 2.55 kV; capillary temperature = 275 ℃; survey MS1 scan = m/z range 350–1800, profile mode, resolution 120,000 FWHM@200 m/z, number of microscan = 1, automatic maximum inject time. Internal calibration was performed using the lock mass for siloxane (445.120024 m/z) as a reference. Data-dependent acquisition Orbitrap survey spectra were scheduled at least every 3 s, with the software determining “Automatic” number of MS/MS acquisitions during this period. The automatic gain control (AGC) target values for FTMS and MSn were 400,000 and 10,000 respectively. The most intense ions (m/z range 350–1800, charge state 2–5) exceeding 50,000 counts were selected for higher-energy collisional dissociation (HCD) ion trap MS/MS fragmentation with detection in centroid mode. Dynamic exclusion settings were: repeat count = 2; exclusion duration = 15 s with a 10 ppm mass window. The ddMS2 IT HCD scan used a quadrupole isolation window of 1.6 Da; rapid scan rate, auto mass range, centroid detection, 1 microscan, automatic maximum injection time, and stepped HCD collision energy 28, 30 and 32%.

### Biological replicate sample preparation using the optimized proteomics workflow

The optimized proteomics workflow used for sample 10 was repeated two more times in order to prepare two additional optimized biological replicate samples (samples 11, 12; both samples from different rabbits). This method was performed as described above with the following key optimizations: (1) NRS was omitted during treponeme isolation; (2) high-speed centrifugation was used for the removal of contaminating proteins during sample preparation; (3) lyophilisation and heat inactivation steps were omitted; and (4) high-pH reversed phase fractionation (24 fractions) was used (Fig. 1). In total 12 biological replicate samples were prepared: samples 1–9 constituted method optimization samples while samples 10–12 constituted samples prepared using the optimized protein preparation method.

### Mass spectrometry parameters and data analyses: protein identifications and validation

Raw files were created by XCalibur 4.3.73.11 (Thermo Scientific) software. Tandem mass spectra were extracted and charge state deconvoluted by Proteome Discoverer version 2.5 (Thermo Scientific). Deisotoping was not performed. All MS/MS samples were analyzed using Sequest (Thermo Fisher Scientific, San Jose, CA, USA; node in Proteome Discoverer 2.5.0.400) containing a customized *T. pallidum* database comprised of all unique protein entries from all National Centre for Biotechnology Information (NCBI) whole proteome annotation revisions of the *T. pallidum* Nichols strain, NCBI reference sequence NC_021490 (18 whole proteome annotation revisions, June 17th 2013–July 4th 2021; https://www.ncbi.nlm.nih.gov/nuccore/NC_021490.2?report=girevhist). This database, “Tpal_06_rabbit_review”, contained 1261 *T. pallidum* sequences, all the reviewed rabbit protein sequences from the UniProt *Oryctolagus cuniculus* proteome, UP000001811, and mass spectrometry common contaminants (https://www.thegpm.org/crap/) (Supplementary Table S1). Database search parameters were as follows: precursor tolerance 10 ppm; MS/MS tolerance 0.6 Da; enzyme specificity was set to trypsin, with a maximum of two missed cleavages allowed; ESI-TRAP instrument type; fixed modification: carbamidomethylation (C); variable modifications: acetyl of the N-terminus and oxidation (M). Scaffold (version Scaffold_5.1.2) (Proteome Software Inc., Portland, OR, USA) was used to validate MS/MS based peptide and protein identifications. Peptide identifications were accepted if they could be established at greater than 95.0% probability by the Percolator posterior error probability calculation^27^. Similar to the previous *T. pallidum* whole proteome mass spectrometry-based study by Osbak et al.^25^, protein identifications were accepted in the present study if they could be established at greater than 95.0% probability and contained at least one identified peptide. In the current study, protein probabilities were assigned by the Peptide and Protein Prophet algorithms^28^. Percolator (as a node in Proteome Discoverer) was used to generate decoy sequences (randomized sequences from the customized *T. pallidum* database, as described above). Similar to the previous *T. pallidum* whole proteome mass spectrometry-based study by Osbak et al.^25^, the false discovery rate (FDR) calculated by Scaffold for confident protein identification was set for less than 5% in the present study. Proteins that contained similar peptides and could not be differentiated based on MS/MS analysis alone were grouped to satisfy the principles of parsimony^29^. Proteins sharing significant peptide evidence were grouped into clusters. Non-identical protein paralog identifications were confirmed via the detection of at least one tryptic peptide that is unique to a single paralog. For identical paralogs (full-length proteins that are identical at the amino acid sequence level e.g., TPANIC_0117 [TprC] and TPANIC_0131 [TprD]), our peptide identification pipeline could not distinguish these as separate protein identifications. The proteome of *T. pallidum* Nichols strain (NCBI reference sequence NC_021490, July 2021 annotation) was used to calculate proteome coverages (964 proteins from predicted protein-coding genes, 15 proteins potentially encoded by 15 genes annotated as “pseudo genes”, and three detected proteins from previous proteome annotations that are not annotated in the July 4th 2021 proteome).

### Mass spectrometry parameters and data analyses: label free quantification

Relative protein abundances in the three *T. pallidum* samples that were prepared using our optimized method (samples 10, 11, 12) (described above and Fig. 1), were determined using label-free quantification (LFQ) based on peptide ion peak intensities as a relative quantitative measure. The LFQ analyses were performed within the FragPipe proteomics pipeline (version 17.1) using the MSFragger proteomic search engine (version 3.4) for protein database searching and peptide identification, Philosopher toolkit (version 4.1.1) for downstream post-processing of MSFragger search results (PeptideProphet and ProteinProphet), and IonQuant for LFQ with FDR-controlled match-between-run (MBR) functionality^30–32^. The RAW spectral files for *T. pallidum* samples 10, 11, and 12 were converted to mzML format with ProteoWizard MS convert (http://proteowizard.sourceforge.net) and loaded into FragPipe and the workflow was configured for LFQ-MBR. The database used for searching and peptide identifications was “Tpal_06_rabbit_review” (described above). MSFragger database search parameters were as follows: precursor mass tolerance − 20 to 20 ppm; fragment mass (MS/MS) tolerance 0.6 Da; enzyme specificity was set to trypsin, with a maximum of two missed cleavages allowed; fixed modification: carbamidomethylation (C); variable modifications: acetylation of the peptide N-terminus and oxidation (M). Peptide and protein identifications were validated and filtered using PeptideProphet and ProteinProphet. Label-free quantification with FDR-controlled match-between-runs in MS1 Quant was performed with the following parameters: Ion quant selected; match between runs enabled; protein quant = MaxLFQ; min ions = 2. All other parameters were set by the LFQ-MBR workflow configuration. Identified proteins were filtered using protein probability (confidence score determined by ProteinProphet from combined evidence from the three samples) and top peptide probability (highest PeptideProphet confidence score from all peptides that map to the protein) thresholds equal to 95% or greater. The MaxLFQ intensity values from the “combined.protein” data output file for the three *T. pallidum* samples were ranked from highest to lowest intensities to determine the relative abundances of the treponemal proteins.

### Functional classification of *T. pallidum* proteins

The genome wide functional annotation tool, eggNOG-mapper version 2.1.9 (http://eggnog-mapper.embl.de/)^33^, was used to assign functional classification to *T. pallidum* proteins. All 1261 *T. pallidum* protein sequences from the “Tpal_06_rabbit_review” database, as described above, were submitted to eggNOG-mapper (default parameters; minimum hit e-value = 0.001, minimum hit bit-score = 60, percentage identity = 40, minimum % of query coverage = 20, minimum % of subject coverage = 20, search against database = eggNOG 5). Functional classifications were based on the “COG_category” (https://www.ncbi.nlm.nih.gov/research/cog) outputs generated for each of the submitted proteins.

## Results

### Optimized protocol for the global analysis of the *T. pallidum* proteome expressed under infection conditions

**Figure 2:**
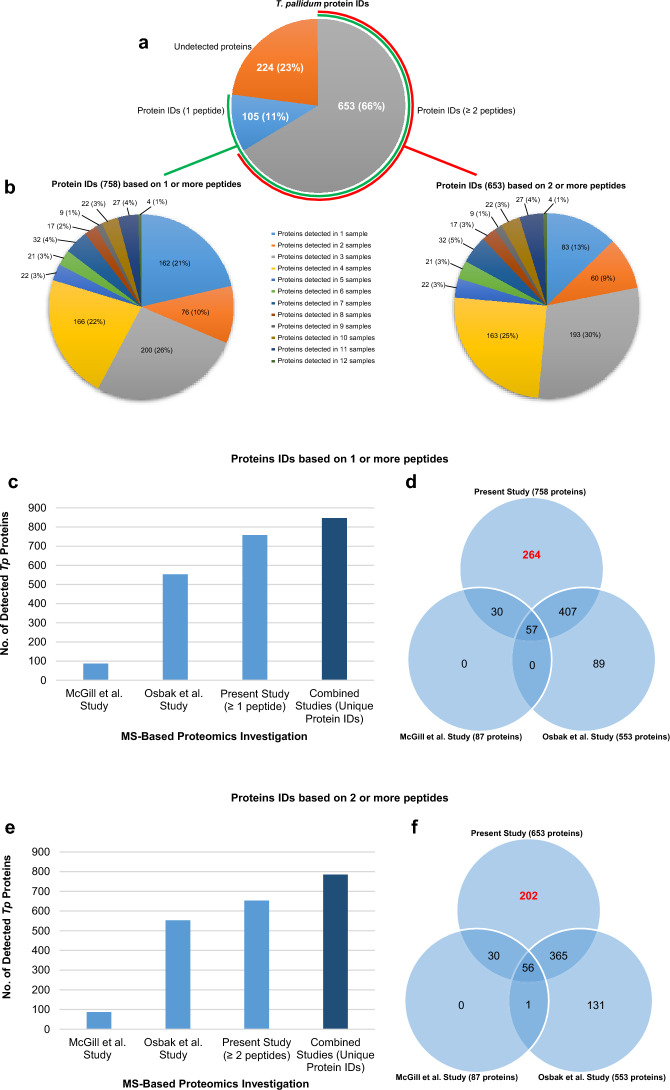
In-depth proteome coverage of in vivo-grown *T. pallidum* and enhancement of the total combined proteome coverage. (**a**) Pie chart depicting the total *T. pallidum* proteome coverage obtained by combining all protein identifications from each of the 12 in vivo-grown biological replicate samples. The total number of treponemal proteins that were identified based on the detection of either one tryptic peptide or at least two tryptic peptides is shown (corresponding proteome coverages are indicated in parentheses). (**b**) Pie charts showing the distribution of protein identification frequencies for the 758 *T. pallidum* proteins that were identified based on the detection of one or more peptides (left) and for the 653 treponemal proteins that were identified based on the detection of at least two peptides (right). Values in parentheses indicate the percentage of *T. pallidum* proteins found in each of the 12 identification frequency groups. (**c**, **e**) Bar graphs showing the total number of *T. pallidum* proteins from in vivo-grown samples that were detected in the present study with at least one (**c**) or at least two (**e**) tryptic peptides and the numbers from previous mass spectrometry-based proteomics investigations (light blue bars). The combined total number of *T. pallidum* proteins identified in the three investigations is also shown (dark blue bar). (**d**, **f**) Venn diagrams showing the total number of shared and exclusive protein identifications in the three mass spectrometry-based proteomics investigations. The total number of *T. pallidum* proteins identified solely in the current study with at least one (**d**) or at least two (**f**) tryptic peptides are highlighted in red text.

To overcome the technical limitations associated with *T. pallidum* experimentation that negatively affect proteome coverage depth, we optimized a workflow specifically for in vivo-grown *T. pallidum.* As shown in Fig. 1, this protocol was comprised of two key steps: (1) *T. pallidum sample* preparation, and (2) mass spectrometry sample preparation. Ten in vivo-grown *T. pallidum* samples (Fig. 1, samples 1–10) were used for method optimization, whereby we investigated the effects of (1) normal rabbit serum (NRS) inclusion during treponeme isolation (Fig. 1a), (2) ultrafiltration (U), high-speed centrifugation (C), lyophilisation, and heat inactivation (HI) in *T. pallidum* sample preparation (Fig. 1b), and (3) high-pH reversed phase peptide fractionation in mass spectrometry sample preparation (Fig. 1c). Our findings demonstrated that the method used for sample 10 provided the highest *T. pallidum* proteome coverage, with a total of 730 treponemal proteins identified (Fig. 1d and Table 1). The optimized method was repeated two more times to obtain three biological replicate samples (samples 10–12). Mass spectrometry analyses of these two samples resulted in the second and third highest proteome coverages in our experiments, respectively (Fig. 1d and Table 1). Detailed mass spectrometry data and Scaffold peptide reports for all 12 in vivo-grown *T. pallidum* samples are presented in Supplementary Table S2.

**Table 1:** Summary of *T. pallidum* proteins identified based on the detection of one or more peptides.

Number of *T. pallidum* proteins detected in each of the 12 biological replicate samples			
In vivo sample	Number of proteins detected	1 peptide for protein ID	2 or more peptides for protein ID
1*	88	14	74
2*	107	8	99
3*	103	16	87
4*	80	20	60
5*	102	14	88
6*	104	12	92
7*	75	41	34
8*	4	3	1
9*	308	54	254
10**	730 (74.3% proteome coverage)	105 (10.7% proteome coverage)	625 (63.6% proteome coverage)
11**	562 (57.2% proteome coverage)	110 (11.2% proteome coverage)	452 (46.0% proteome coverage)
12**	557 (56.7% proteome coverage)	103 (10.5% proteome coverage)	454 (46.2% proteome coverage)

Total coverage of the *T. pallidum* proteome			
In vivo samples	Number of proteins detected	1 peptide for protein ID	2 or more peptides for protein ID
1–12 combined	758 (77.2% proteome coverage) (596 detected in ≥ 2 biological replicates) (520 detected in ≥ 3 biological replicates)	105 (10.7% proteome coverage)	653 (66.5% proteome coverage) (570 detected in ≥ 2 biological replicates) (510 detected in ≥ 3 biological replicates)

Proteome coverage of *T. pallidum*: this study and previous studies^24, 25^			
In vivo samples	Number of proteins detected	1 peptide for protein ID	2 or more peptides for protein ID***
1–12 combined and previous studies^24, 25^	847 (86.25% proteome coverage) (264 proteins identified only in the present study)	62 (6.31% proteome coverage)	785 (79.94% proteome coverage) (202 proteins identified only in the present study)

Detection of *T. pallidum* Miniproteins of unknown function			
In vivo samples	Number of miniproteins detected	1 peptide for protein ID	2 or more peptides for protein ID
1–12 combined	28 (41.2% miniprotein coverage) (19 miniproteins identified only in the present study)	8 (11.8% miniprotein coverage)	20 (29.4% miniprotein coverage) (13 miniproteins identified only in the present study)

Detection of hypothetical proteins and proteins of unknown function			
In vivo samples	Number of proteins detected	1 peptide for protein ID	2 or more peptides for protein ID
1–12 combined	175/264 total proteins detected (66.3% coverage) 98/162 “hypothetical proteins” (60.5% coverage) 17/35 DUF domain proteins (48.6% coverage) 60/67 “poorly annotated proteins” (89.6% coverage)	29 (11.0% coverage) 14 (8.6% coverage) 5 (14.3% coverage) 10 (14.93% coverage)	146 (55.3% coverage) 84 (51.9% coverage) 12 (34.3% coverage) 50 (74.63% coverage)

Detection of known or predicted OMPs			
In vivo samples	Number of proteins detected	1 peptide for protein ID	2 or more peptides for protein ID
1–12 combined	27 (79.4% known/predicted OMP coverage) (11 OMPs identified only in the present study)	9 (26.5% known/predicted OMP coverage)	18 (52.9% known/predicted OMP coverage) (5 OMPs identified only in the present study)

Detection of putative pathogenesis-related proteins (PRPs)			
In vivo samples	Number of proteins detected	1 peptide for protein ID	2 or more peptides for protein ID
1–12 combined	28 (82.4% coverage) (7 PRPs identified only in the present study)	4 (11.8% coverage)	24 (70.6% coverage) (6 PRPs identified only in the present study)

*Method development samples; **optimized method samples; ***protein identifications based on two or more peptides in the present study.

### Total coverage of the *T. pallidum* proteome

A total of 758 treponemal proteins were identified in the 12 *T. pallidum* samples, representing 77% total proteome coverage (Fig. 2a and Table 1); 596 were detected in at least two biological replicate samples (Fig. 2b and Supplementary Table S3). This analysis is similar to the previous in vivo-grown *T. pallidum* global proteomics study performed by Osbak et al.^25^, which based protein identifications on the detection of at least one peptide. A high level of confidence of these protein identifications was ensured in the present study via the use of mass spectrometry validation algorithms and software (Peptide Prophet and Protein Prophet in Scaffold, and Percolator)^27–29^. A total of 653 *T. pallidum* proteins were identified based on the detection of at least two peptides, representing 66% total proteome coverage (Fig. 2a and Table 1); 570 were detected in at least two biological replicate samples (Fig. 2b and Supplementary Table S3).

Of note, 62 *T. pallidum* proteins (6.0% total proteome coverage) were detected in nine or more biological replicate samples (Fig. 2b and Supplementary Tables S3 and S4). Most of these proteins are annotated with functions that are essential for the basic functioning of *T. pallidum* (“housekeeping proteins”) (Supplementary Table S4 and Supplementary Fig. S1). A list of all the proteins detected in each of the 12 samples is presented in Supplementary Table S5.

The 224 annotated *T. pallidum* proteins that were not detected in the present study are listed in Supplementary Table S6. Almost 40% of these undetected proteins were annotated in the proteome as “hypothetical proteins” or as DUF (Domain of Unknown Function) domain-containing proteins, and almost 50% were either not assigned a function or classified as “function unknown” using COG (Clusters of Orthologous Genes) analysis (Supplementary Table S6 and Supplementary Fig. S2). Sixty-three (28% of the undetected proteins) were miniproteins comprised of less than 150 amino acids^34^. The expected reasons for lack of detection of these 224 proteins in our study are described below.

### Enhanced proteome coverage of *T. pallidum*

Two previous mass spectrometry-based proteomics studies identified a combined total of 587 *T. pallidum* proteins (60% proteome coverage)^24, 25^. When these results were combined with the findings from the present study, a total of 847 *T. pallidum* proteins were identified (86% proteome coverage; includes proteins identified based on the detection of a single peptide in the present study) (Fig. 2c, Table 1, and Supplementary Table S7). As shown in Fig. 2d, Table 1, and Supplementary Table S7, 264 *T. pallidum* proteins were identified only in the current study, which increased the combined proteome coverage by 27%. When protein identifications were based on the detection of two or more peptides in the present study, a combined total of 785 *T. pallidum* proteins were identified (80% proteome coverage) (Fig. 2e, Table 1, and Supplementary Table S7). Using the two peptide identification criteria, 202 *T. pallidum* proteins were identified only in the current study, which increased the combined coverage of the treponemal proteome by 21% (Fig. 2f, Table 1, and Supplementary table S7).

The list of 135 *T. pallidum* proteins (from Nichols strain, NCBI reference sequence NC_021490, July 2021 annotation) not detected in the present study, or in either of the two previous mass spectrometry-based proteomics studies^24, 25^, is presented in Supplementary Table S8. This group contained 43 proteins with functions assigned in the proteome as “hypothetical” and 12 assigned as DUF domain-containing proteins. Notably, 50 of the undetected proteins were miniproteins comprised of 150 amino acids or less. Small protein size (average length of 101 amino acids) was likely an important contributing factor that prevented detection of these 50 miniproteins, as outlined below. In addition, over 50% of the 135 undetected proteins were either not assigned a function or classified as “function unknown” using COG analysis (Supplementary Fig. S3). In summary, these investigations have identified over 50% of all previously undetected *T. pallidum* proteins, and increased the combined proteome coverage from 60% to almost 90%.

### Identification of *T. pallidum* miniproteins

The *T. pallidum* proteome contains 68 open reading frames (ORFs) predicted to encode miniproteins (comprised of 150 amino acids or less) of unknown function, two of which were confirmed to be expressed at the RNA and/or protein levels^25, 34^ and were capable of exhibiting antimicrobial peptide (AMP) activities^34^. In the present study, we detected expression of 28 of these predicted 68 miniproteins. Out of the 28 detected miniproteins, 19 were identified solely in this work, including 4/6 of the previously identified top-ranked predicted AMPs^34^ (Supplementary Fig. S4a, Table 1, and Supplementary Table S9). When the results of the two previous proteomics studies^24, 25^ are combined with the findings from the present study, a total of 34/68 *T. pallidum* miniproteins have been confirmed to be expressed at the protein level during infection (Supplementary Fig. S4a and Supplementary Table S9), thereby increasing the combined proteome coverage for *T. pallidum* miniproteins of unknown function from 22% to 50%.

### Identification of hypothetical proteins and proteins of unknown function

*Treponema pallidum* is a phylogenetically distinct bacterium with ~ 30% of all predicted protein-coding genes having no known orthologs or assigned functions^15, 35^. A search of the *T. pallidum* proteome identifies 264 proteins of unknown functions, including 162 “hypothetical proteins”, 35 DUF domain-containing proteins, and 67 proteins with annotations that provide minimal insight into potential functions (Supplementary Table S10). In the current study we detected 98/162 of these hypothetical proteins. Out of these 98 detected hypothetical proteins, 45 were identified solely in the present study (Supplementary Fig. S4b, Table 1, and Supplementary Table S10). Of the 64 remaining hypothetical proteins that were undetected in our study, 28 are miniproteins comprised of 150 amino acids or less. Seventeen of the 35 DUF domain-containing proteins were detected in the current study, 12 of which had not been detected previously. A total of 60/67 proteins with annotations that give minimal insight into potential functions (“poorly annotated proteins”, Table 1) were also identified; 21 of these 67 proteins were identified solely in the present study. Overall, we detected 175/264 treponemal proteins of unknown function in the current study, with 78 detected for the first time (Supplementary Fig. S4b, Table 1, and Supplementary Table S10). When combined with the two previous mass spectrometry studies^24, 25^, 207 of the 264 *T. pallidum* proteins of unknown function have now been confirmed to be expressed at the protein level during infection.

### Identification of known and predicted surface-exposed OMPs

As shown in Table 2, a search of the literature identified 34 *T. pallidum* surface-exposed OMPs that have either been experimentally confirmed or predicted to be OMPs^10, 24, 25, 36–51^. Our analyses identified 27 of these 34 known/predicted OMPs (Tables 1 and 2), 11 of which were detected for the first time in the present study, including the *T. pallidum* repeat (Tpr) protein family members^9^ TprK (TPANIC_0897) and TprL (TPANIC_1031), and the vascular adhesin TPANIC_0751^52, 53^. In total, we detected 7/12 Tpr proteins, all seven of which have been reported in the literature as predicted/known surface-exposed OMPs^25, 36–38, 41–43, 46, 48^ (Table 2). Including the current study, 10/12 Tpr proteins have now been detected in treponemes isolated from infections via mass spectrometry-based proteomics studies (Table 2)^25^. When combined with the results from the two previous mass spectrometry studies^24, 25^, a total of 31/34 predicted and known *T. pallidum* surface-exposed proteins have now been detected at the protein level during infection.

**Table 2:** Mass spectrometry-based detection of predicted/known OMPs from in vivo-grown *T. pallidum.*

OMP locus tag	NCBI functional annotation	MS detection	References
TPANIC_0009	Hypothetical protein, TprA	ND	^43^
TPANIC_0011	Major outer sheath C-terminal domain-containing protein, TprB	Osbak^25^	^25, 42, 43^
TPANIC_0117*^#^	Major outer sheath N-terminal domain-containing protein, TprC	Present, Osbak^25^	^25, 37, 42, 43^
TPANIC_0126	Hypothetical protein	Present, Osbak^25^	^10, 25, 45, 47, 50^
TPANIC_0131*^#^	Major outer sheath N-terminal domain-containing protein, TprD	Present, Osbak^25^	^25, 37, 42, 43^
TPANIC_0155	M23 family metallopeptidase	Present, Osbak^25^	^25, 38, 39, 43^
TPANIC_0313	Major outer sheath N-terminal domain-containing protein, TprE	Present, Osbak^25^	^25, 43^
TPANIC_0316	Hypothetical protein, TprF	ND	^38, 43^
TPANIC_0324/325	Translocation/assembly module TamB domain-containing protein	Osbak^25^	^25, 43, 47^
TPANIC_0326*	Outer membrane protein assembly factor BamA	Present, Osbak^25^	^25, 38, 40, 43, 44, 47, 50^
TPANIC_0421	Tetratricopeptide repeat protein	Present, Osbak^25^	^25, 43, 50^
**TPANIC_0479**	DUF2715 domain-containing protein	Present	^47^
**TPANIC_0483**	Fibronectin type III domain-containing protein	Present	^38^
**TPANIC_0515**	LPS-assembly protein LptD	Present	^10, 47, 50^
**TPANIC_0548**	UPF0164 family protein	Present	^10, 43, 47, 50^
**TPANIC_0557**	DUF1007 family protein	Present	^38^
TPANIC_0620	Major outer sheath N-terminal domain-containing protein, TprI	Present, Osbak^25^	^25, 36, 38, 42, 43, 46^
TPANIC_0621	Major outer sheath N-terminal domain-containing protein, TprJ	Present, Osbak^25^	^25, 43^
TPANIC_0698	DUF2715 domain-containing protein	ND	^47^
**TPANIC_0733**	Outer membrane beta-barrel protein	Present	^10, 47, 50^
TPANIC_0751*	Vascular adhesin/metalloprotease pallilysin	Present	^38, 49, 51^
TPANIC_0855	Hypothetical protein	Present, Osbak^25^	^25, 43^
TPANIC_0856	UPF0164 family protein	Present, Osbak^25^	^10, 38, 47, 50^
TPANIC_0858	UPF0164 family protein	Present, Osbak^25^	^10, 25, 43, 47, 50^
TPANIC_0859	UPF0164 family protein	Osbak	^10, 47, 50^
TPANIC_0865	UPF0164 family protein	Present, Osbak^25^	^10, 25, 43, 47, 50^
TPANIC_0897*	MSP porin, TprK	Present	^41, 43^
TPANIC_0923	PEGA domain-containing protein	Osbak^25^	^25^
**TPANIC_0952**	Alpha/beta fold hydrolase	Present	^38^
**TPANIC_0966**	Hypothetical protein, TolC	Present	^10, 47, 50^
TPANIC_0967	Hypothetical protein, TolC	Present, Osbak^25^	^10, 47, 50^
TPANIC_0968	Hypothetical protein, TolC	Present, Osbak^25^	^47, 50^
TPANIC_0969	Hypothetical protein, TolC	Present, Osbak^25^, McGill^24^	^10, 25, 43, 47, 50^
**TPANIC_1031**	Major outer sheath N-terminal domain-containing protein, TprL	Present	^43, 48^

*Proteins with experimental evidence indicating *T. pallidum* surface exposure. ND: protein not detected.

^#^In the present study, the protein identified as TPANIC_0117 may be TPANIC_0117, TPANIC_0131, or both (both proteins contain the identified peptides).

McGill: Proteins detected in McGill et al. study (2010). Osbak: Proteins detected in Osbak et al. study (2016). Present: Proteins detected in the present study (Present: protein identification based on the detection of one tryptic peptide).

Proteins in bold font: Proteins from in vivo-grown *T. pallidum* that were identified only in the present study.

In addition to the NCBI functional annotations, Tpr family member names are also included where applicable.

### Identification of putative pathogenesis-related proteins

A search of the literature revealed 34 *T. pallidum* proteins that were previously identified as potential pathogenesis-related proteins based on proteome-wide tertiary structure modeling and treponemal genome sequencing studies^35, 50, 54, 55^. In the current study, we detected expression of 28 of these 34 proteins (Tables 1 and 3). When combined with the results from the two previous mass spectrometry studies^24, 25^, a total of 31/34 *T. pallidum* proteins with predicted pathogenesis-related functions have now been shown to be expressed during infection.

**Table 3:** Mass spectrometry-based detection of predicted pathogenesis-related proteins from in vivo-grown *T. pallidum.*

Locus tag	Functional Annotation (Nichols strain NC_021490, July 2021 annotation)	Potential functions from previous studies* ** ***	MS detection
TPANIC_0020***	VWA domain-containing protein	TgMIC2 (*Toxoplasma gondii* micronemal protein 2 A/I domain)	Present, Osbak^25^
TPANIC_0027*	Hemolysin family protein	Putative hemolysin (HlyC)	Osbak^25^
TPANIC_0028*	Hemolysin family protein	Putative hemolysin (HlyC)	ND
TPANIC_0126***	Hypothetical protein	Outer membrane protein W (*E. coli*)	Present, Osbak^25^
TPANIC_0134***	Hypothetical protein	Bacterial sialidases/neuraminidases	Present
TPANIC_0225***	Leucine-rich repeat domain-containing protein	Leucine-rich repeat surface proteins	Present
TPANIC_0246***	VWA domain-containing protein	TRAP protein (*Plasmodium vivax*)	Osbak^25^
TPANIC_0262**	Crp/Fnr family transcriptional regulator	PrfA (*Listeria monocytogenes* virulence factor transcriptional regulator)	Present, Osbak^25^
TPANIC_0399*	Flagellar M-ring protein FliF	Type 3 (virulence-related) secretory pathway protein (FliF)	Present, Osbak^25^
TPANIC_0401*	Flagellar assembly protein FliH	Type 3 (virulence-related) secretory pathway protein (FliH)	Present, Osbak^25^
TPANIC_0402*	Flagellar protein export ATPase FliI	Type 3 (virulence-related) secretory pathway protein (FliL)	Present, Osbak^25^
TPANIC_0421***	Tetratricopeptide repeat protein	PknD (*Mycobacterium tuberculosis* serine/threonine protein kinase, extracellular domain)	Present, Osbak^25^
TPANIC_0544***	SpnA family nuclease	SmcL (*Listeria ivanovii* Sphingomyelinase-C)	Present, Osbak^25^
TPANIC_0579***	Hypothetical protein	YenC2 (*Yersinia entomophaga* ABC toxin; BC component)	Osbak^25^
TPANIC_0594***	DUF2147 domain-containing protein	HP1028 (*Helicobacter pylori* lipocalin)	Present
TPANIC_0598***	Hypothetical protein	BamB (*Moraxella catarrhalis* Beta barrel assembly machinery protein B)	Present, Osbak^25^
TPANIC_0625***	Hypothetical protein	BamD (Beta barrel assembly machinery protein) (*Rhodothermus marinus*, *E. coli*)	Present, Osbak^25^
TPANIC_0649*	Hemolysin family protein	Putative hemolysin (TlyC)	Present, Osbak^25^
TPANIC_0714*	Flagellar biosynthesis protein FlhA	Type 3 (virulence-related) secretory pathway protein (FlhA)	Present, Osbak^25^
TPANIC_0715*	Flagellar biosynthesis protein FlhB	Type 3 (virulence-related) secretory pathway protein (FlhB)	Present, Osbak^25^
TPANIC_0733***	Outer membrane beta-barrel protein	NspA (*Neisseria* surface protein A)	Present
TPANIC_0783***	Hypothetical protein	BamB (*E. coli* beta barrel assembly machinery protein B)	Present
TPANIC_0789***	Outer membrane lipoprotein-sorting protein	LolA (*P. aeruginosa* outer- membrane lipoprotein carrier/localization protein)	Present, Osbak^25^, McGill^24^
TPANIC_0854***	SpoIIE family protein phosphatase	Bacterial sialidases/neuraminidases	Present, Osbak^25^
TPANIC_0862**	FKBP-type peptidyl-prolyl cis–trans isomerase	Mip (*Legionella pneumophila* macrophage infectivity potentiator protein)	Present, McGill^24^
TPANIC_0911***	FlhB-like flagellar biosynthesis protein	EscU (*E. coli* type 3 secretion system protein)	ND
TPANIC_0928***	Hypothetical protein (previous proteome annotation; not annotated in July 2021)	SurA (*E. coli* chaperone)	Present, Osbak^25^
TPANIC_0936*	Hemolysin family protein	Putative hemolysin	ND
TPANIC_0966***	Hypothetical protein	TolC (*E. coli* outer membrane channel protein)	Present
TPANIC_0967***	Hypothetical protein	TolC (*E. coli* outer membrane channel protein)	Present, Osbak^25^
TPANIC_0968***	Hypothetical protein	TolC (*E. coli* outer membrane channel protein)	Present, Osbak^25^
TPANIC_0969***	Hypothetical protein	TolC (*E. coli* outer membrane channel protein)	Present, Osbak^25^, McGill^24^
TPANIC_1033**	Patatin-like phospholipase family protein	VipD (*Legionella pneumophila* phospholipase effector protein)	Present, Osbak^25^
TPANIC_1037*	Hemolysin III family protein	Putative hemolysin III (HlyIII)	Present

*Proteins identified as potential virulence factors in *T. pallidum* genome sequencing and comparison studies^35, 54, 55^.

**Proteins identified as novel virulence factor candidates (proteins previously annotated with non-virulence related functions) using whole proteome structure modeling (highest-ranking predicted virulence-related structural homolog listed)^50^.

***Proteins identified as novel virulence factor candidates (proteins of unknown function) using whole proteome structure modeling (highest-ranking predicted virulence-related structural homolog listed)^50^.

ND: Protein not detected.

McGill: Proteins detected in McGill et al. study (2010). Osbak: Proteins detected in Osbak et al. study (2016). Present: Proteins detected in the present study (Present: protein identification based on the detection of one tryptic peptide).

Proteins in bold font: Proteins from in vivo-grown *T. pallidum* that were identified only in the present study.

### Identification of *T. pallidum* proteome annotation errors

To facilitate the mass spectrometry analyses, we generated a customized *T. pallidum* database for mass spectrometry-based protein identifications that contained all *T. pallidum* proteins that had been annotated in the proteome by NCBI Prokaryotic Genome Annotation Pipeline (PGAP) software from June 2013-July 2021. This approach identified three proteins annotated as “hypothetical proteins” that were removed from the July 2021 proteome annotation (TPANIC_RS02075, TPANIC_RS04705, and TPANIC_0928), one of which was detected in the present study for the first time (Table 4 and Supplementary Fig. S5a). We also identified three proteins annotated with prematurely truncated N-termini (Table 4 and Supplementary Fig. S5b–5d). The premature N-terminal truncation of TPANIC_0765 (ATP-dependent zinc metalloprotease, FtsH) is predicted to remove one of two transmembrane helices, however, this failed to alter the predicted inner membrane location (data not shown), a locale that is consistent with the *E. coli* homolog of this protein^56^. We also detected the in vivo expression of 6/15 proteins from ORFs that were annotated by NCBI as “pseudo” genes (non-coding ORFs), including Tp0897 (TprK) (Table 4). This finding is in agreement with previous studies that have demonstrated expression of Tp0897/TprK at the RNA^57, 58^ and protein levels using opsonophagocytosis assays^41^, enzyme-linked immunosorbent assays (ELISA)/antibody-binding assays^59, 60^, and mass spectrometry-based analysis of in vitro-cultured *T. pallidum*^61^.

**Table 4:** *T. pallidum* NCBI proteome annotation errors.

Locus tag	Functional annotation	NCBI Proteome Annotation Error (in Nichols strain NC_021490, July 2021 annotation)	MS detection
TPANIC_RS02075*	Hypothetical protein	Protein deleted from proteome	Present, Osbak^25^
TPANIC_0928*	Hypothetical protein	Protein deleted from proteome Protein replaced by TPANIC_RS05505 (“Pseudo” hypothetical protein, homologous to TPANIC_0928 N-terminus) and TPANIC_RS05510 (hypothetical protein, homologous to TPANIC_0928 C-terminus) TPANIC_0928 peptide (R)ELSFEDAVATGSTK(V) detected in 3 samples (peptide is not present in TPANIC_RS05505 or TPANIC_RS05510)	Present, Osbak^25^
TPANIC_RS04705*	Hypothetical protein	Protein deleted from proteome	Present
TPANIC_0446	(E)-4-hydroxy-3-methylbut-2-enyl-diphosphate synthase	Incorrectly truncated N-terminus (MNQRDERAARQPEEKV peptide truncated at N-terminus of TPANIC_0446 [latest version, WP_010881894]) TPANIC_0446 (former version, WP_014342797) peptide (K)VDSSAGVSPCNSPYGSGLLDVPLK(L) detected in 2 samples (peptide is not present in the latest version of TPANIC_0466)	Present, Osbak^25^
**TPANIC_0535**	Hypothetical protein	Incorrectly truncated N-terminus (MSAAWVGNMDKGVMVRLAEVEDAAAVLVEKAQEQAQR peptide truncated at N-terminus of TPANIC_0535 [latest version, WP_010881982]) TPANIC_0535/TP_RS02625 (former version, WP_014342464) peptide (R)LAEVEDAAAVLVEK(A) detected in 5 samples (peptide is not present in the latest version of TPANIC_0535)	Present
TPANIC_0765**	ATP-dependent zinc metalloprotease FtsH	Incorrectly truncated N-terminus (113 amino acids**** truncated at N-terminus of TPANIC_0765 [latest version, WP_187145723]) TPANIC_0765 (former version, WP_014342822) peptide (K)QSDDSSDPFGFFK(F) detected in 2 samples (peptide is not present in the latest version of TPANIC_0765)	Present, Osbak^25^, McGill^24^
TPANIC_0007	DUF3798 domain-containing protein	“Pseudo” (non-coding annotation)	Present, Osbak^25^
TPANIC_RS01255***	Hypothetical protein	“Pseudo” (non-coding annotation) (Previously annotated as TP_0248 in the Osbak et al. study)	Present, Osbak^25^
TPANIC_0533***	V-type ATP synthase subunit I	“Pseudo” (non-coding annotation)	Present, Osbak^25^
TPANIC_0813***	Hypothetical	“Pseudo” (non-coding annotation)	Present, Osbak^25^
**TPANIC_0897**	MSP porin (TprK)	“Pseudo” (non-coding annotation)	Present
TPANIC_0993***	Septal ring lytic transglycosylase RlpA family protein	“Pseudo” (non-coding annotation)	Present, Osbak^25^

*Proteins have been re-added in the Nichols strain (March 2023 Nichols NC_021490 annotation [TPANIC_RS02075 locus tag corresponds to TPANIC_0425 in the March 2023 annotation; TPANIC_RS04705 locus tag corresponds to TPANIC_RS05630 in the March 2023 annotation which has an incorrectly truncated N-terminus based on the results from the present study]).

**Protein has been re-annotated with the correct N-terminus (March 2023 Nichols NC_021490 annotation).

***Proteins have been re-annotated as coding proteins (March 2023 Nichols NC_021490 annotation).

****Truncated amino acids: MCFFAAPCIPPQRTSLSCAVRLSHSLSTFHLLFVYHGPACPRALQKGALTEMNTRYKQSDDSSDPFGFFKFSPRPQKGPSSSRERPPRRNSRKVLSLVLLALCALLALANHFL.

McGill: Proteins detected in McGill et al. study (2010). Osbak: Proteins detected in Osbak et al. study (2016). Present: Proteins detected in the present study (Present: protein identification based on the detection of one tryptic peptide).

Proteins in bold font: Proteins from in vivo-grown *T. pallidum* that were identified only in the present study.

Eight of the 12 proteome errors detected in the present study have been revised and re-annotated since our analyses were performed (March 2023 proteome annotation revision). One of these deleted proteins (TPANIC_RS04705) that had been re-added in the March 2023 version of the proteome (locus tag re-named TPANIC_RS05630) was shown in the present study to have a prematurely truncated N-terminus based on the detection of a peptide (EVFEEELSALEHR [Leucine corresponds to the start site in the 2023 annotation]) (Supplementary table S2). In addition, four proteins of unknown function that had been removed from the latest *T. pallidum* proteome annotation (TPANIC_0126a, TPANIC_0135, and TPANIC_1030) or not annotated in the Nichols strain used in this study (TPANIC_0922) were each detected in a previous mass spectrometry-based proteomics study^25^. These findings confirm the usefulness of in-depth proteomic analysis for clarifying uncertainties associated with *T. pallidum* genome/proteome annotations (Table 4).

### Global relative abundances of *T. pallidum* proteins

The relative abundance of proteins detected in the three optimized *T. pallidum* samples (10, 11, 12) were determined using label-free quantification (LFQ) based on peptide ion peak intensities (MaxLFQ intensity values; higher intensity corresponds to higher protein abundance) (Supplementary Table S11). A summary of all high-abundant proteins with LFQ intensity values greater than the mean average in each of the three samples is provided in Supplementary Table S12. These higher abundance proteins were found to be predominantly involved in metabolism, homeostasis and survival, chemotaxis and motility, and protein translation (Supplementary Table S12 and Supplementary Fig. S6). Less than 10% were found to be proteins of unknown function (“hypothetical proteins” and DUF domain-containing proteins) (Supplementary Table S12); these included three miniproteins TPANIC_0084, TPANIC_0847, and TPANIC_0777 (Supplementary Table S9), one of which has been predicted to function as an AMP (TPANIC_0847)^34^. Surprisingly, the miniprotein of unknown function, TPANIC_0214, which was previously identified as the most abundant protein in *T. pallidum*^25^ and as an AMP candidate^34^, was not identified as a highly abundant protein in the present study. In addition, only three proteins predicted to have pathogenesis-related functions via tertiary structure modeling (Table 3)^50^ were identified as higher abundant proteins; TPANIC_0225 (structural homolog of leucine rich repeat surface proteins), TPANIC_0789 (structural homolog of outer membrane lipoprotein carrier/localization protein, LolA), and TPANIC_0862 (structural homolog of macrophage infectivity potentiator, MIP). None of the quantified proteins from the assembled list of 34 known or predicted treponemal surface-exposed OMPs (Table 2) were assigned LFQ intensity values higher than the mean average in each of the three samples (Supplementary Tables S11 and S12). In addition, the relative abundance rankings for known/potential OMPs ranged from: (1) 173–459 out of 465 (sample 10); (2) 205–405 out of 446 (sample 11); and (3) 317–421 out of 438 (sample 12) (Supplementary Fig. S7a–c). In these studies, frequency of protein detection correlated with protein abundance and protein functions required for pathogen survival. For example, the 62 most frequently detected *T. pallidum* proteins, which were detected in nine or more of the 12 biological replicate samples (Supplementary Tables S3 and S4 and Supplementary Fig. S1), had high relative abundances and were annotated as possessing functions essential for survival of *T. pallidum* (Supplementary Tables S11 and S12). These global protein abundance analyses provide both insight into *T. pallidum* protein expression patterns during infection and a baseline for comparative studies of in vitro-/in vivo-grown *T. pallidum*.

## Discussion

The in-depth proteomic characterization of a biological system provides knowledge important for understanding its primary bioactive molecules, including the global expression profile, and data pertaining to the structure, function, and regulation of the biological system^62^. In the present study, we optimized a proteomics workflow specific for *T. pallidum* that focused on the preparation of mass spectrometry-compatible samples. Sample preparation is an important, yet often overlooked, aspect of experimentation that greatly impacts the outcome of proteomic analyses involving complex biological systems, such as *T. pallidum* grown under infection conditions. In the present study, optimizing the sample preparation methodology allowed for an improved global expression profile of in vivo-grown *T. pallidum* and attainment of knowledge regarding the expression status of proteins with potential roles in the survival and pathogenesis of *T. pallidum* during infection, including OMPs.

Surface-exposed OMPs are targets of *T. pallidum* protective antibodies, however, these proteins are relatively rare compared to the OMPs of other more conventional bacteria^10–12^. The optimized workflow reported in this study detected, for the first time, expression of 11 OMPs from *T. pallidum* grown under infection conditions (in vivo-grown *T. pallidum*). Literature reports provide evidence that several of these detected proteins play key roles in *T. pallidum* pathogenesis and have been identified as current syphilis vaccine candidates, including TPANIC_0751^51, 63, 64^, TPANIC_0897 (TprK)^16, 65, 66^, and TPANIC_1031 (TprL)^48^. Most of the detected Tpr proteins are predicted to be surface-exposed OMPs and have been shown to elicit an immune response during experimental syphilis infection^67–69^. Some of the Tpr proteins also undergo antigenic^16–18^ and phase variation^19, 20^, which may facilitate immune evasion^9^. Surface-exposed OMPs that are targeted by protective antibodies, demonstrate inter- and intra-strain amino acid sequence conservation, and are expressed at the protein level are three important considerations in the design of effective recombinant-based protein vaccines^8, 9, 70, 71^. Thus, the OMP expression findings reported here have relevance for syphilis vaccine design, in that they confirm the in vivo expression of current or future vaccine candidates.

In the present investigation, less than 10% of proteins identified with high relative abundances were identified as proteins of unknown function. Predicted pathogenesis-related proteins and OMPs were also found to be of low relative abundance, consistent with previous microscopy-based studies that showed the rarity of *T. pallidum* surface-exposed OMPs^10–12^. Indeed, the low expression levels of these proteins may be one of the immune evasion strategies used by *T. pallidum* during infection.

The current study also provides the first published report of proteome annotation errors in *T. pallidum*. These findings improve *T. pallidum* proteome annotation and contribute to the broader field of research focused on improving genome and proteome annotations. The genome/proteome of the *T. pallidum* strain used in the current study has been revised 20 times over 10 years. Interestingly, frequent genome/proteome re-annotations are not restricted to *T. pallidum*; the genomes/proteomes for the reference strains *Escherichia coli* O157:H7 str. Sakai DNA (NCBI accession number NC_002695.2) and *Mycobacterium tuberculosis* H37Rv (NCBI accession number NC_000962.3) have been revised 70 and 76 times, respectively. This finding highlights the highly dynamic nature of automated pipelines used in bacterial genome and proteome annotation. Similar to our study, a proteogenomics study of *Helicobacter pylori* identified four novel ORFs that were not identified during annotation, and facilitated correction of the amino acid sequences of six annotated proteins^72^. Regarding more complex eukaryotic genomes, it has been reported that genome errors affect up to 50% of all the coding sequences in non-human primate proteomes^73^. These findings illustrate the importance of considering the potential for annotation errors when selecting protein targets for experimentation and subunit vaccine design, and of integrating experimental proteomics findings into genome/proteome annotation pipelines for improved annotation accuracy.

There are limitations associated with the current study. First, the analyses were performed using the Nichols strain of *T. pallidum*. This strain was first isolated in 1912 from the cerebrospinal fluid (CSF) of an individual with secondary syphilis^74^, and has since been passaged continuously in rabbits. Although this laboratory reference strain remains virulent, future studies using recently isolated clinical strains will provide valuable comparative data on the global protein expression profile of low passage strains. Second, the small size and positive charge of many *T. pallidum* miniproteins^34^ may contribute to their lack of detection by mass spectrometry. Specifically, trypsin cleaves after positively-charged arginine and lysine residues; consequently, as protein size decreases and charge increases, the number of peptides generated from trypsin digestion that fall within the detection range of most mass spectrometers (~ 6–50 amino acids) also decreases. Thus, the possibility exists that these undetected miniproteins are expressed during *T. pallidum* infection, but their physiochemical properties limit detection by mass spectrometry and will require other experimentation to confirm expression at the protein level. Third, the majority of predicted/known OMPs were unable to be semi-quantified using LFQ, most likely due to their low abundance. Future studies focused on the development of *T. pallidum* OMP enrichment techniques and/or label-based quantitative mass spectrometry methods could be performed to obtain improved quantification of treponemal OMPs. Fourth, since rabbits are outbred, rabbit-to-rabbit variability (e.g. different immune responses) can lead to differing *T. pallidum* protein expression levels among samples. Finally, mass spectrometry studies can only provide information on detected proteins and cannot provide insight in the absence of protein detection.

In conclusion, our findings can be used to inform rational syphilis vaccine design by confirming the expression of vaccine candidates during infection, an essential step in any vaccine development pipeline. Our results also identified protein forms and ORFs that, although being incorrectly annotated or unannotated in the *T. pallidum* proteome, are expressed during infection. In addition to the possibility that these “novel” proteins could constitute syphilis vaccine candidates, these findings highlight the under-reported, but important, issue of incorrect genome/proteome annotations across all species. Finally, the optimized sample preparation reported in the present study provides a workflow for obtaining protein expression profiles from complex, host-derived samples of clinical *T. pallidum* strains.

### Supplementary Information

Supplementary Figures.

Supplementary Table S1.

Supplementary Table S2.

Supplementary Table S3.

Supplementary Table S4.

Supplementary Table S5.

Supplementary Table S6.

Supplementary Table S7.

Supplementary Table S8.

Supplementary Table S9.

Supplementary Table S10.

Supplementary Table S11.

Supplementary Table S12.

## Data Availability

All mass spectrometric raw data files and Scaffold search engine files are publicly available on the MassIVE repository at https://massive.ucsd.edu/ProteoSAFe/dataset.jsp?task=ce1736216d154959b0dc932061e315e8 under the identifier MSV000092028 (ProteomeXchange identifier PXD042479).
