# Supplementary material for: Deep proteome coverage advances knowledge of Treponema pallidum protein expression profiles during infection
Source: Sci Rep. 2023 Oct 25;13:18259. doi: 10.1038/s41598-023-45219-8 (PMC10600179; doi:10.1038/s41598-023-45219-8)
Supplement: Supplementary file 1 — Supplementary Figures. [file 41598_2023_45219_MOESM1_ESM.pdf]

Deep Proteome Coverage Advances Knowledge of *Treponema pallidum* Protein Expression Profiles During Infection

Simon Houston[1], Alloysius Gomez[1], Andrew Geppert[1], Azad Eshghi[2], Derek S. Smith[2], Sean Waugh[1], Darryl B. Hardie[2], David R. Goodlett[1,2], and Caroline E. Cameron[1,3]*

Supplementary Information (Figs. S1-S7)

Supplementary Figure S1

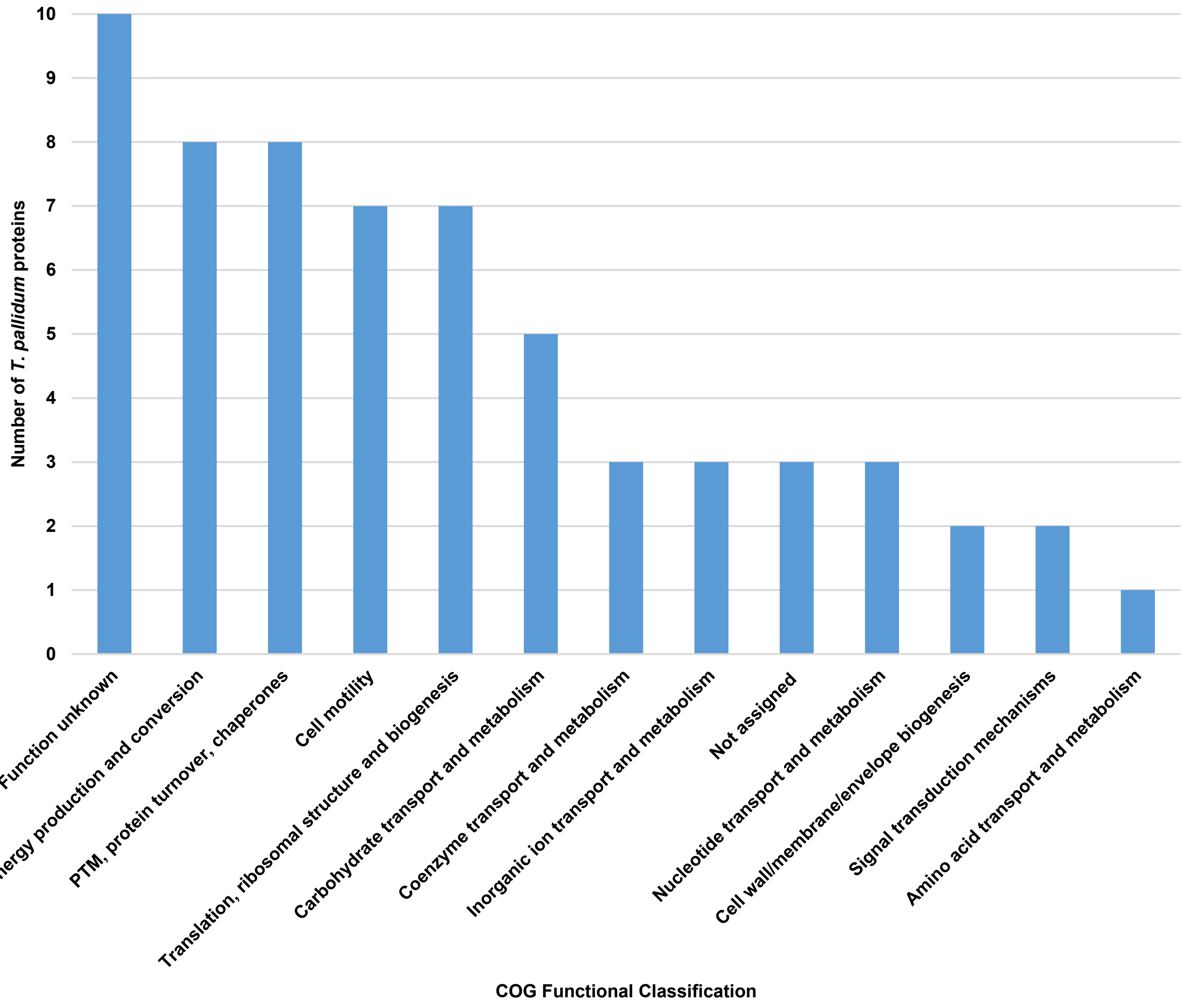

Supplementary Figure S1. Functional classification of *T. pallidum* proteins detected in more than nine samples. Bar chart showing the distribution of 62 *T. pallidum* proteins detected in 9 - 12 of the in vivo-grown biological replicate samples, based on COG functional classifications. COG functional classifications were generated by the eggNOG-mapper tool. PTM – post-translational modification. Not assigned – proteins where eggNOG-mapper was unable to assign functional classifications.

Supplementary Figure S2

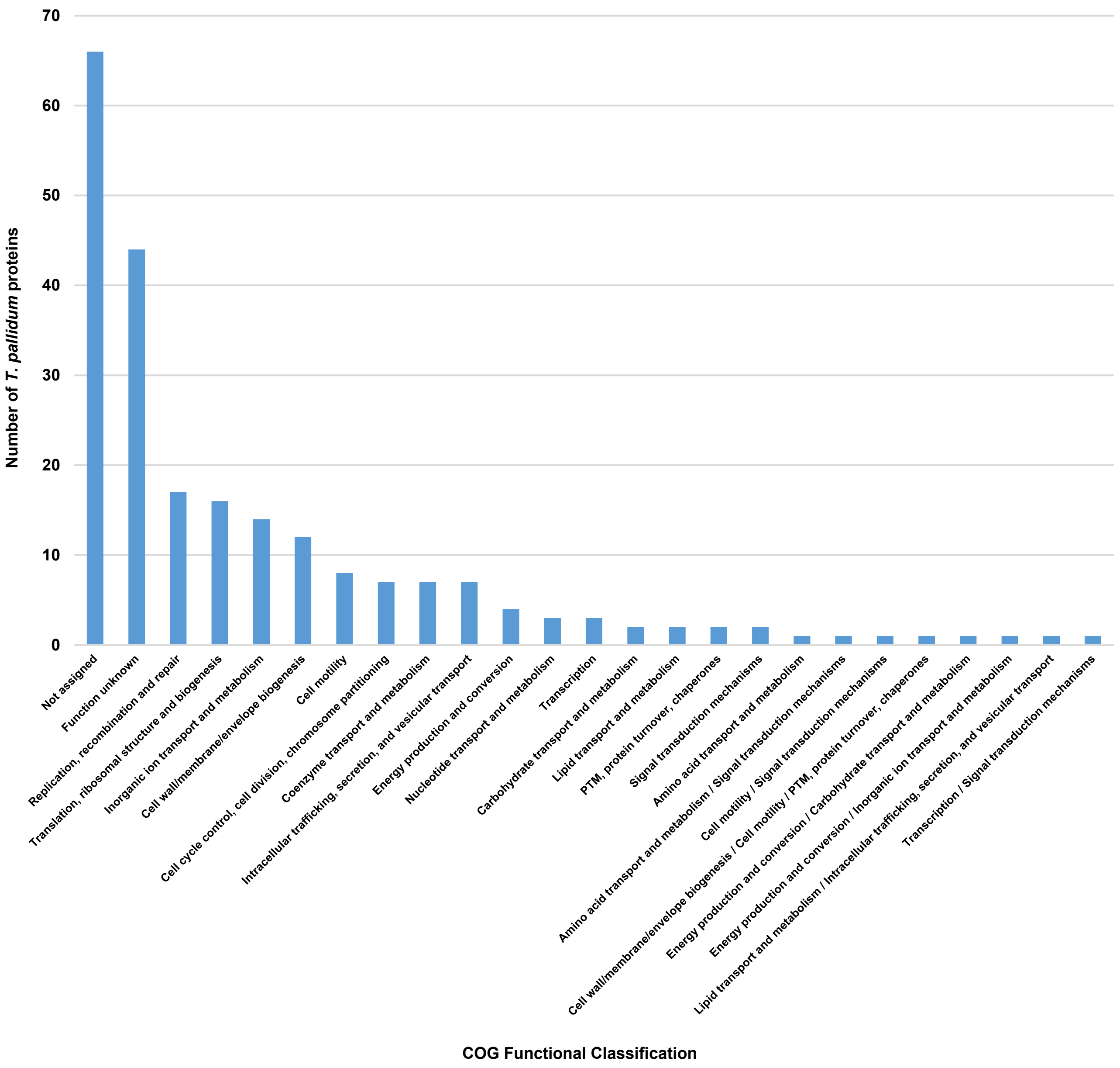

Supplementary Figure S2. Functional classification of undetected *T. pallidum* proteins in the present study. Bar chart showing the distribution of 224 undetected *T. pallidum* proteins based on COG (Clusters of Orthologous Genes) functional classifications generated by the eggNOG-mapper tool. PTM – post-translational modification. Not assigned – proteins where eggNOG-mapper was unable to assign functional classifications.

Supplementary Figure S3

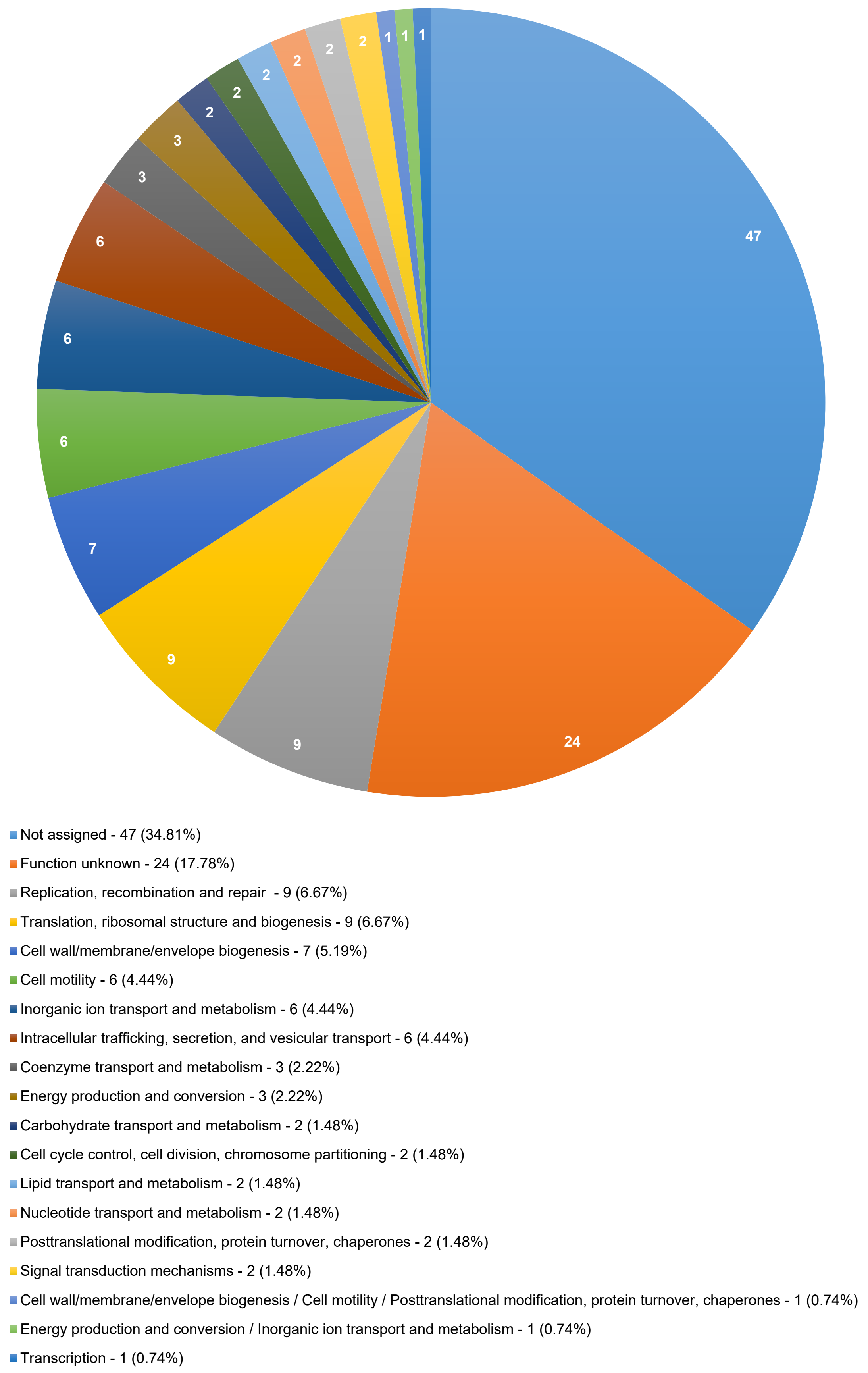

Supplementary Figure S3. Functional classification of undetected treponemal proteins from past and present global proteomics studies. Pie chart depicting the COG-based functional classification distribution of 135 *T. pallidum* proteins that have not been detected in the present or previous [24,25] global proteomics studies from in vivo-grown *T. pallidum*. COG functional classifications were generated by the eggNOG-mapper tool. The number of proteins comprising each COG classification category are shown in the pie chart and in the figure key. The figures shown in parentheses (figure key) correspond to the percentage of undetected proteins in each of the COG functional categories (out of a total of 135 proteins). Not assigned – proteins where eggNOG-mapper was unable to assign functional classifications.

Supplementary Figure S4

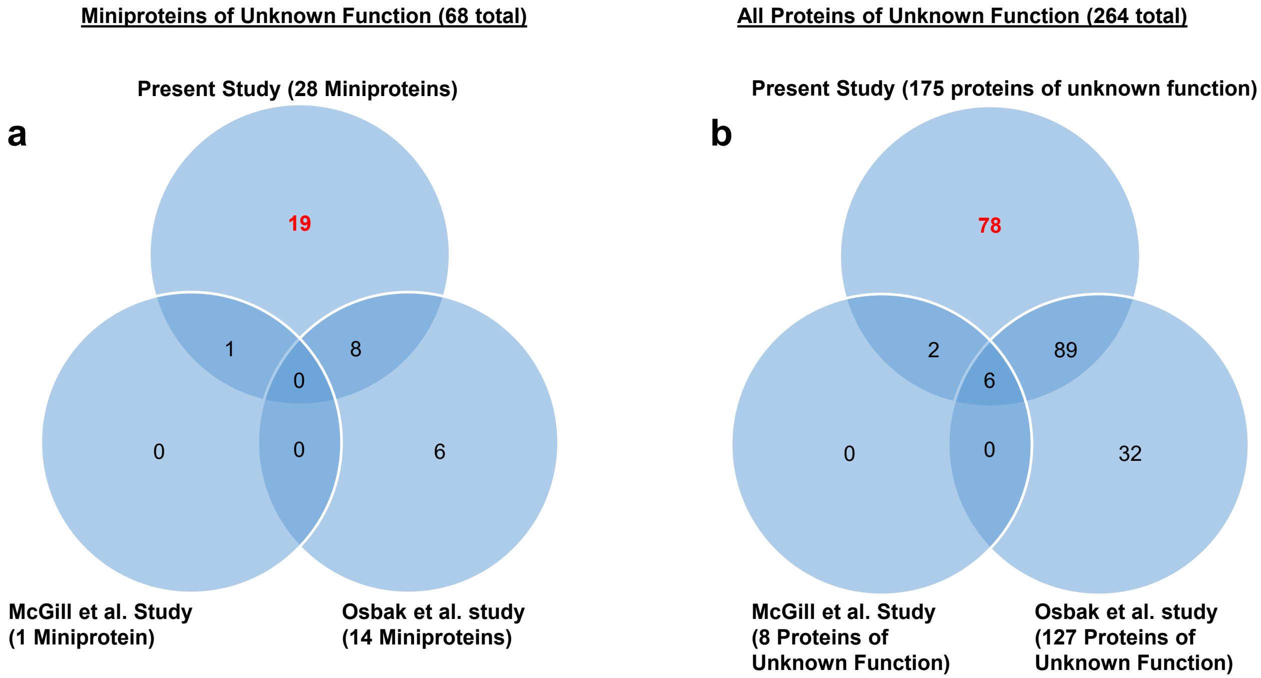

Supplementary Figure S4. Proteome coverage corresponding to *T. pallidum* proteins of unknown function. a, Venn diagram showing the total number of shared and exclusive identifications corresponding to miniproteins (≤ 150 amino acids) of unknown function (68 in the proteome) from in vivo-grown *T. pallidum* in the three mass spectrometry-based proteomics investigations. The total number of *T. pallidum* miniproteins identified exclusively in the current study (based on the detection of at least one tryptic peptide per identification) is highlighted in red text. **b,** Same as **a**, with the exception that the data relates to the identification of all (no size limitation) *T. pallidum* proteins of unknown function (from a total of 264 proteins identified in the proteome).

Supplementary Figure S5

a

TPANIC_0928

MKSFYFGRGALASTGGCVVLAVAALMFVFLPALSGGGHGHVRLGSWGGVALTNAPDSLFSQQYSALAQTAERYNLYPKDAQEREGMDRRVLRAAFRAA
VVQLAGTEQTKQSGFTLSEELLDREVLSFYADADGTYSPQRYQGTPEHVRLSQRKKMRDSLLSDQYLYHLFGKETGRGGLKLNSRELRFVQDMAKKERSFRY
VVLGEERFPAERVSAYGKEHAHLFTLHHLSLLTYSSEEDARRTARALEKRELSFEDAVATGSTKVGTDATGKMTHSYRSDVNEFFPDAQDLDTVLRTAVGALS
PVVKIERGFAIVRTDAEPSAPDFSDAATRERVFAHMARVERGTIERFLEEEAHTFSTRAKQGGFTAAAQSLNLDVHTS**RS**FPINFGNVDVLPALPRQSDPPLAR
IAYDEKFFSTAFALLPGQVSDPLILDSSVVLLQLHEEKSVDESVLATTGDSYAHHVRTWYPGYPLALLNTAKMPWAQESVVDTILAHPSFKDTFDNIFRR

b

TPANIC_0446

MNQRDERAARQPEEKVDSSAGVSPCNSPYGSGLLDVPLKLRPRASHYRARSLVIGGKEHVRALPLGGDAPIPIQTMWKEPLIGADLQSIVDRLLELEQ
LGCDVVRFAVPDRESAELFVALCARTRMPLVADIHFDYRLALRCMDGPVAKVRINPGNIGVRERVRAVVEKARATGTALRIGVNTGSLPGVVKRAVA
ARYADGMQSVNARAEALVQTAFAEAAYLDQLHFDRVVLSLKASTVAETVRANELFAQQSDIPLHLGVTEAGPLVSGIVKSTLAFSQLLSRNIGATVRV
SLSDSMEHEVLAAREILAECGKRAGGVRLVSCPRCGRIGFDVHAFVRRWQKELFSLKKDITVAVMGCVVNGPGEGKHADLGISGAEDSVIFFKRGKIV
RRIQVRDLCADERTRIIDAAFKEELSSL

c

TPANIC_0535

MSAAWVGNMDKGVMVRLAEVEDAAAVLVEKAQEQAQRMRESAEAAGETLVKEAEGSVVEAQEQAWHEACARIDAQRREACGQLERELSVLEEDVE
RFEAFLDGFFFG

d

TPANIC_0765

MCFFAAPCIPPQRTSLSCAVRLSHSLSTFHLLFVYHGPACPRALQKGALTEMNTRYKQSDDSSDPFGFFKFSPRPQKGPSSSRERPPRRNSRKVLSLVLLALC
ALLALANHFLFSRALRIIPFSEFKDRIASGEIVKVVVGSPYFVGYTSARPAPSARGFSLLSEREAPTYHAIGVLSDSFLSMLDERQVVYSIKPRERNYLIETFQYLFP
LLILFFVWRFFFKRMASNVSGLGSSIFSAGHARSAAVEEGKVTTRFADVAGVDEAKEELMEVVDFLKFPKKYTEIGGKIPRGVLLVGPPGTGKTLLARAVAGE
ASVPFFRISGSDFIEMFVGIGASRVRDLFKQAREKAPGIIFIDELDAIGKSRLNAIHSNDEREQTLNQLLVEMDGFDNTTGLILLAATNRPDVLDPALLRPGRFD
RQVCVDRPDLKGREAILRIHAQNVKLAPEVDLKAVARITGGYSGADLANVVNEAALLAVRSGRAQVIETDLDEAVEKTMIGLQKKSRVIREEERRIIAYHETG
HALAGTFTKGADKVHKITIIPRGTSALGYTFHIPEDDRHIVTEQQLLAEVDVLLSGRAAEFVAFGEVSTGAGNDISRATDIVRKMITDYGMSEKFQNVALTRR
GTGYLAEPQLAREYSECTQQYVDEEVARVLAERYRAVVALLTEKKELLEYIATRLLERETIERDEFEEVIRCEHDLEGLKKRLSQQQEGASAPAQGQSPPS

Supplementary Figure S5. *T. pallidum* proteome annotation errors. a, Amino acid sequence of TPANIC_0928. This protein was incorrectly deleted from the NCBI *T. pallidum* proteome (Nichols strain NC_021490, July 2021 annotation) and replaced with two shorter proteins, one homologous to the N-terminus of TPANIC_0928 (TPANIC_RS05505, homologous sequence shown in green text) and one homologous to the C-terminus of TPANIC_0928 (TPANIC_RS05510, homologous sequence shown in blue text). We detected a TPANIC_0928 peptide (ELSFEDAVATGSTK) (underlined) in three *T. pallidum* biological replicate samples that is not present in either TPANIC_RS05505 or TPANIC_RS05510. Residues in black text are not present in either TPANIC_RS05505 or TPANIC_RS05510 but present in TPANIC_0928. Amino acid sequences of **b,** TPANIC_0446, **c,** TPANIC_0535, and **d,** TPANIC_0765 were annotated with incorrect N-terminal truncations in the NCBI *T. pallidum* proteome. Peptides from previous NCBI annotated versions (pre July 2021) of each of the three proteins were detected in the present study (underlined). Blue arrows indicate the start sites of the incorrectly truncated protein versions in the NCBI July 2021 proteome annotation. The extended N-termini corresponding to the previously annotated versions of each protein is highlighted in red font.

Supplementary Figure S6

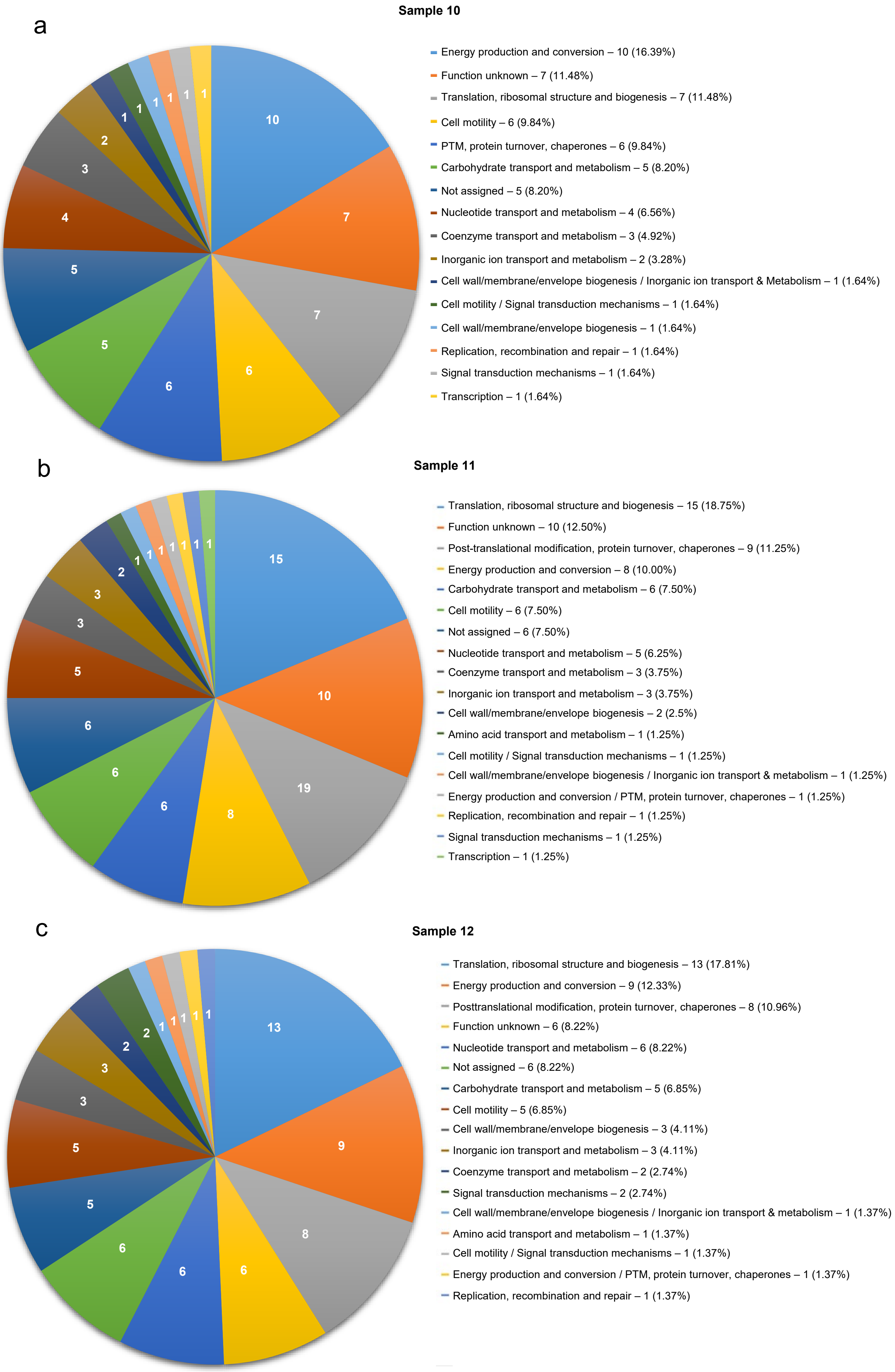

Supplementary Figure S6. Functional classification of high-abundant *T. pallidum* proteins. a-c, Pie chart depicting the COG-based functional classification distribution of 61 high-abundant (LFQ intensity values greater than the mean average) *T. pallidum* proteins detected in sample 10 (**a**), 80 detected in sample 11 (**b**), and 73 detected in sample 12 (**c**). COG functional classifications were generated by the eggNOG-mapper tool. The number of proteins comprising each COG classification category are shown in the pie chart and in the figure key. The numbers shown in parentheses (figure key) correspond to the percentage of detected, high-abundant proteins in each of the COG functional categories. PTM – post-translational modification. Not assigned – proteins where eggNOG-mapper was unable to assign functional classifications.

Supplementary Figure S7

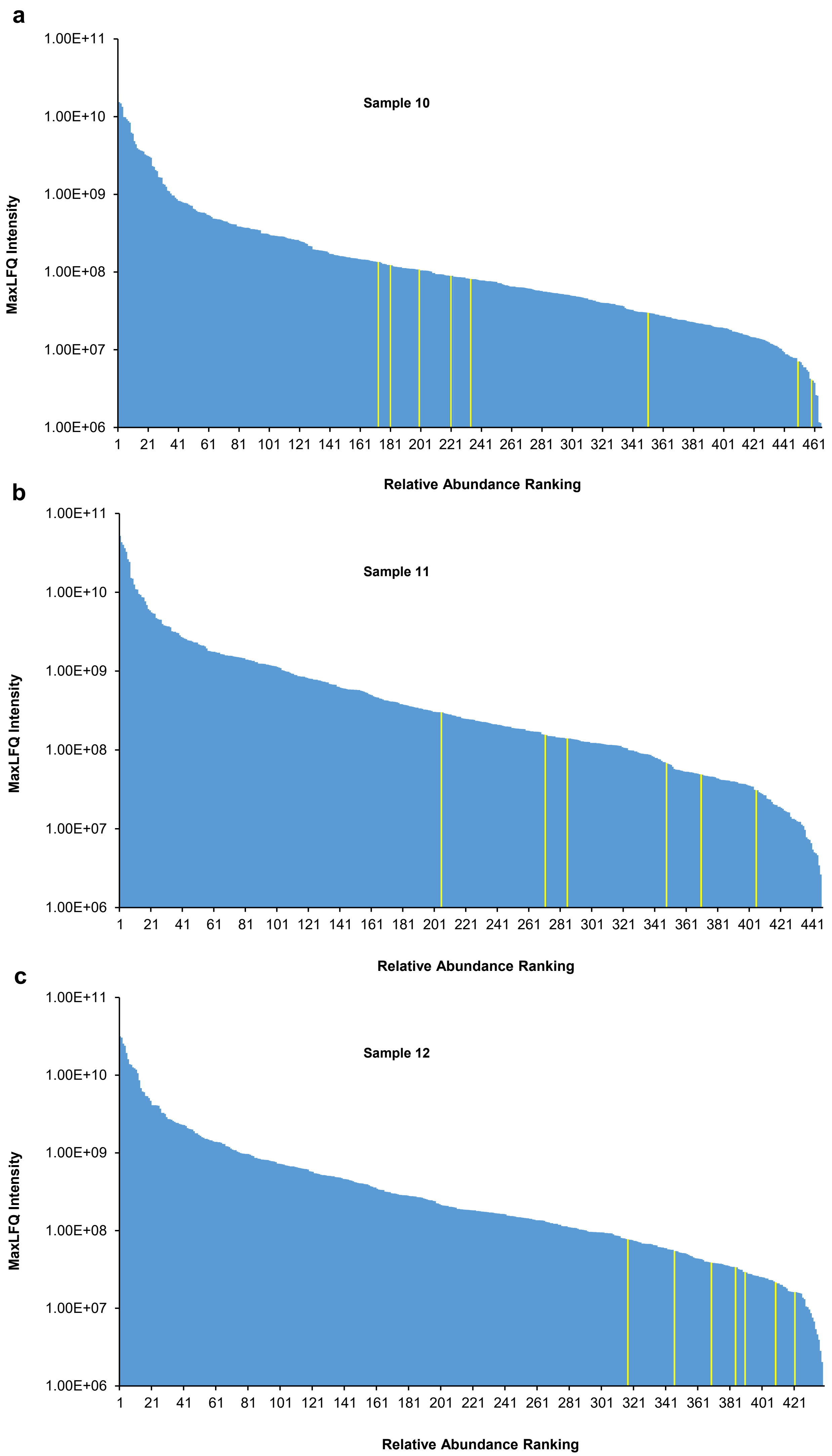

Supplementary Figure S7. Relative abundances of *T. pallidum* OMPs. a-c, Graph showing the relative abundances of 465 treponemal proteins (ranked from highest to lowest relative abundance [x-axis]), including eight known/predicted OMPs (yellow highlighting), that were semi-quantified based on peptide ion peak intensities (MaxLFQ intensity values [y-axis]) in sample 10 (**a**), 446 treponemal proteins (including six known/predicted OMPs) in sample 11 (**b**), and 438 treponemal proteins (including seven known/predicted OMPs) in sample 12 (**c**). Higher MaxLFQ intensity corresponds to higher protein abundance.
